# Relationship between Immunosuppressive Medications Adherence and Quality of Life and Some Patient Factors in Renal Transplant Patients in Iran

**DOI:** 10.5539/gjhs.v6n4p205

**Published:** 2014-04-16

**Authors:** Maryam Shabany Hamedan, Jaleh Mohamad Aliha

**Affiliations:** 1 School of Nursing and Midwifery, Department of Medical Surgical Nursing, Iran University of Medical Science, Tehran, Iran

**Keywords:** medication adherence, immune – system suppressors, quality of life, renal transplant

## Abstract

**Background & Aim:**

About organ transplant, immunosuppressive medications adherence is a critical issue, because non-adherence to these medications causes rejection, reduces quality of life and increases treatment cost and mortality rate. Among these, the quality of life is deemed very important to evaluate treatment result and also it can be useful for discovering non adherence. The aim this study was to assess the relationship between medication adherence and quality of life and some patient factors in renal transplant patients.

**Methods:**

The study was a descriptive-correlational design and was done on renal transplant patients over 18 who had undergone surgery for over 3 months, and were inclined to participate. Sample size was 230 people and sampling was convenience. Quality of life questionnaire in renal transplant patients and Immunosuppressant Therapy Adherence Scale were filled by patients and the data was analyzed by SPSS15 software.

**Results:**

It showed that the mean score of quality of life in renal transplant patients was 21.65±4.03 and 57.8% of them did not adhere to immunosuppressive medications. Results of correlation between scores of immunosuppressive medication adherence and Quality of life showed that there were significant correlation in 3 dimensions of 4: health performance (p≤0.0001 & rETA=0.23), social-economic (p=0.001 & rETA=0.15), psychological-spiritual (p=0.011 & rETA=0.15), also logistic test showed significant relationship between immunosuppressive medication adherence and number of transplantation (β=1.04, p= 0.048).

**Conclusion:**

According to the results, health care providers i.e. nurses must note to medication adherence as a health enhancement factor while treating and educating to these patients.

## 1. Background

Nowadays, renal transplant is a satisfying and proven method of treating advanced chronic kidney diseases. There has been tens of thousands of transplants in the world and in some centers, 50 to 100 kidney transplant operations are performed annually (Fauci et al., 2008). In Iran, the kidney transplant rate is 24 per million which is 1-5 per million in developing countries and 20-40 per million in advanced countries. Iran with over 20,000 annual operations ranks 1^st^ in the region (Support Association of Kidney Patients online, 2007).

Renal transplant releases the patient from many restrictions of dialysis (Rambod, Shabany-Hamedan, Shokrpour, Rafii, & Mohammad alliha, 2011) but is not the final treatment of kidney anomaly. This operation is in fact a follow-up of treatment with its own side-effects and potential impacts (Phipps, Neighbors, & Green, 2007). Survival after transplant emphasizes adherence to immunosuppressive therapy (Kugler et al., 2007).

The powerful combination of immunosuppressive therapy gives excellent short-term results and guarantees transplant acceptance with little potential of acute rejection (Hansen, Seifeldin, & Noe, 2007; Massey et al., 2013). It is very clear that advantage of medication regimen is realized when patient makes logical and precise use of prescription (Marcén, 2009; Osterberg & Evert Koop, 2005).

The renal transplant receivers must follow the immunosuppressive medication regimen for a long time so the rejection is prevented and mortality is reduced. Therefore a high degree of adherence to immunosuppressive therapy is essential (Denharynck & Belgien, 2006).

Non adherence to immunosuppressive therapy is an important issue in renal transplant patients. Almost 20% of renal transplant patients do not adhere to these groups of medications. The non adherence involvement in transplant rejection is 16% and in acute rejection is 20% (Osterberg & Evert Koop, 2005). Also past research showed there are defects in adherence to immune –suppressor system intake in receivers of the organ. Instead of dangers caused by non adherence, 20- 25% of adults who receive heart, liver & kidney transplant do not adhere to adherence to their body immunosuppressive therapy and it is and finally this will lead to reduced quality of life. Also, the side –effects of using immunosuppressive therapy affect various aspects of life (Mann, 2004). Measuring quality of life in clinical studies makes patient feel more intimate to the doctor and health service providers. This makes patient aware of their disease and the conditions that lead to health, getting acquainted to advantages & disadvantages of various treatment and emphasizing the patient role in choosing treatment method (Donlad, 2003).

Measuring quality of life toget access to essential statistics for policy-making and planning has many applications in social / clinical goals (Donlad, 2003). Since on one side, it is vital to take clinical decisions for the needy person and on the other, in taking social decisions for planning and following a policy (Molnar-Varga, 2011). Here, the nurses are very effective in implementing strategy for improving adherence since they are present in all healthcare scenes and intimacy with the people. They can ask patient about consumed drugs, their effect on their quality of life and identifying & educating patients who do not adhere to their immune – system suppressors, can prevent transplant defect of even patient death (Fredericks et al., 2008).

## 2. Materials & Methods

This study was a descriptive correlational designand was conducted to assess the relationship between immunosuppressive medication adherence and quality of life in renal transplant patients.

The study population consisted of all transplant patients with over 3 months after transplant and over 18 years. The sample size at 90% reliability was 230 persons. The research samples were selected consistently (convenience) from among research population, i.e. all qualified patients with the required capacity participated in the research. Sampling took 2.5 months. Sample exclusion criteria were people with mental/bodily disabilities, transplantpatients who had less than three months. The research was down at the council of advocacy of renal disorder patients and its affiliate treatment clinic.

For assessing immunosuppressive medication adherence was used of Immunosuppressant Therapy Adherence Scale (ITAS). In this questionnaire, 12 score means full adherence and below that means non adherence. This tool was designed based on Likert 4-options scale with 4 questions (in the last 3 months how often did you: forgot to take their immunosuppressant therapy or IST medications, careless about taking their IST medications, stopped taking their IST medications because they felt worse, and missed taking their IST medications for any reason) and is matched with selecting the appropriate option by the patient. The options are never=3, seldom=2, often=1, always=0.

Patient selects one option based on the way he/she adherence to immunosuppressive medication.

The criteria for immunosuppressant therapy adherence is 12, i.e. the person must select *never* to be considered adhered. Toclarifythe reasons of non-adherence in the last question, researcher listed some of them and patients were asked to check mark them. Questionnaire may be filled in 3-5 minutes.

The second questionnaire relates to the quality of life in renal transplant patients and it is composed of 2 sections: satisfaction & importance. Each section has 35 questions and each question is scored relative to its rating. The questionnaire is rated by 6-options Likert type scale in the first section as 6=very satisfied to 1=very dissatisfied and in the second section as 1=very unimportant to 6= very important. The 2 sections have similar statements. This tool measured 4 dimensions in quality of life: health/performance, socioeconomics, psychological/spiritual, family. The questionnaire was applied as interview or as filled by the person (self-report) and takes 15 minutes. Validity of both tools was determined through formal content validity method. So that after translating questionnaires from English to Persian and again back to English and removing any back-translated distortion, 10 experts of Midwifery & Nursing Faculty of Iran Medical Science University, checked and verified this tool. The internal consistency of the questionnaire for adherence to immunosuppressive therapy was calculated through Coefficient Alfa =0.78. This tool was calculated by Chisholm et al. (2005) using Coefficient Alfa =0.81. Also, the reliability of the questionnaire for quality of life was calculated through Coefficient Alfa =0.8 and this tool was calculated by France & Powers using Coefficient Alfa =0.9.^.^

The researcher began the study after obtaining the permit of Iran Midwifery & Nursing Faculty Ethical Committee. At first, the researcher attended the council of advocacy of renal disorder patients and its affiliate treatment clinic and after full explanation of research goals and emphasis on confidentiality and obtaining written consent, collected the samples. Two questionnaires of adherence to medication and quality of life in renal transplant patients were completed by the samples. To answer research questions, the Chi-Square and Logistics Regression was used relative to application.

## 3. Results

Data was analyzed by SPSS15 software and showed that the meanparticipants age was 41.69 years (SD=12.174) with min age 18 and max age 68. The frequency percentage of men and women was respectively 55.2 and 44.8. The most frequency of marital status in both sexes was for married individuals (72.2%) and the least was for the divorced (4.3%). Most of the participants had intermediate degree (28.7%) and minorities were in university level (10.4%). Highest frequency distribution of occupational status was among housewives (34.8%) and least among students (0.9%). Most participants were of economic middle class (50.6%) and a few were of higher class (3.5%). Most patients were urban (88.7%) and some (10.9%) were living in rural areas. The most frequency of length of time for using immunosuppressive therapy (time since the transplant) was 5.00 years (SD=3.9) and the most frequency type of immunosuppressive medication regimen was Cellcept-Sandimmune(cyclosporine)-Prednisone (76.52%) and the least was monotherapy regimen that is, only Cellcept (0.4%).

Also based on number of prescribed medications by practitioner, the most frequency was 3-medications (88.7%), and the least was 1 medication (0.4%). Most of the patients (66.5%) citedthat used other medications except immunosuppressive medications (for example: Medications for hypertension, hyperlipidemia, seizures and anti depressant and so on), also they had forgot number, type and name of other medications and did not say precisely. and the most frequency of the number of using immunosuppressive therapyper day was 2 times (71.7) and the least was 3 times (11.3). The mean number of using immunosuppressive therapy per day was 2.45 times (SD =0.768). The most frequency of transplant operation was first time operation (90%). Most frequency of donor types was alive-stranger (93%) and the least was alive-family (2.6 %). Most frequency of distribution of chronic disease was related to *no disease* option equal to 39.6%. Most frequency of distribution of chronic disease type was hypertension (47.96%) and then diabetes (14.47%) and the least was related to other disease (colic, asthma, G6PD, lupus) equal to 1.8%. Most patients were non adherence (57.8) and mean score of quality of life was 21.65(SD=4.03).most of the patients had disireble quality of life (71.3%).

In assessing the relationshipbetween adherence to immunosuppressant medication therapy with some of the more relevant parametersprobably, it was revealed that adherence has significant relation with age (p=0.049, X^2^=8.873), The time since transplant (p=0.041, X^2^=9.948) and the number of renal transplant (p=0.036, X^2^=4.376) (Table 1) Since the frequency distribution is based on the adherence and non-adherence, the sum of percentages adjusted horizontally. Because there is no space and vertical table seemed more appropriate here. Also Table 2 shows that most percentage (sum of percentage) related to non adherence is for question one that is forgot to take the immunosuppressive medications and for other reasons following the response to question 4, price of medication is more than the others.

**Table 1:** Relationship between adherence and variables

Adherence immunosuppressive drug variables	Adherence		Non adherence		Chi-Square statistic	

	N	%	N	%	(X^2^)	P
Age						
30≥	19	40/4	28	59/6	7/87	0/049*
31-40	29	18	44	71		
41-50	34	53/1	30	46/9		
51≤	26	45/6	31	54/4		
Sex						
Woman	41	39/8	62	60/2	0/42	0/51
Man	56	44/1	71	55/9		
Marital status						
Single	13	33/3	26	66/7	3/25	0/35
Married	73	44	93	56		
Divorced	6	60	4	40		
Widowed	5	33/3	10	66/7		
Occupational status						
Employed	34	45/3	41	54/7		
Pension	19	48/7	20	51/3		
Unemployed	14	41/2	20	58/8		
Housewife/men	29	36/3	51	63/8		
Student	1	50	1	50	2/20	0/45
Educational level						
Illiterate	12	38/7	19	61/3		
Elementary	25	39/1	39	60/9		
Secondary	18	40	27	60		
Intermediate	34	51/5	32	48/5		
University& higher	8	31/3	16	68/7	3/62	0/45
Frequency of medication useduring the day						
2	71	43	94	57		
3	12	46/2	14	53/8		
≥ 4	14	35/9	25	64/1	0/84	0/65
Economical status						
Poor	51	48.1	55	51.9		
Fair	43	37.1	73	62.9		
Good	3	37.5	5	62.5	2.84	0.09
The time since transplant						
≤1	20	57/1	15	42/9		
2-5	46	37/7	76	62/3		
6-9	21	46/7	24	53/3		
10-15	10	47/6	11	52/4		
16-21	0	0	100	7	9/94	0/041*
Type of immunosuppressive medication regimen						
Cyclosporine-cellcept- Prednisone	71	40/3	105	59/7		
Azathioporine-Cyclosporine-Prednisone	17	51/5	16	48/5		
Prednisone -Cyclosporine	2	28/6	5	71/4		
Cyclosporine- Cellcept	6	46/2	7	53/8		
Cellcept	1	100	0	0	1/42	0/49
Number of prescribed immunosuppressive medications						
1	1	100	0	0**		
2	10	42.3	15	57.7		
3	86	42.2	118	57.8	0.0	0.99
number of using immunosuppressive therapy per day						
2	71	43	94	57		
3	12	46.2	14	53.8		
≥ 4	14	35.9	25	64.1	0.84	0.65
Chronic disease						
yes	59	64.9	80	87.8		
No	38	41.8	53	58.2	0.619	0.89
Number of transplant						
First transplant	92	44/4	115	21/7	4/37	0/036*
Second transplant	5	55/6	18	87/3		
Type of transplant						
alive-familiar	2	33/3	4	66/7		
alive-strange	93	43/5	121	56		
Cadaver	2	20	8	80	2/08	0/149
Quality of life						
Relatively desirable	16	16/5	50	37/6		
desirable	81	83/5	83	62/4	12/20	*<0/0001

* There is significant relationship

** 2 columns of one & two medications merged for Chi-square test.

**Table 2:** Reason for non-adherence


**Reason for non-adherence**	**Never(3)**	**Seldom(2)**	**Often(1)**	**Always(0)**
Immunosuppressant Therapy Adherence Scale				
1-forgot to take	138(60%)	56(24.3%)	26 (11.3%)	10(4.3%)
2-careless about taking	144(62.6%)	51(22.2%)	28(12.2%)	7(3%)
3-stop taking to feeling worse	209(90.9%)	5(2.2%)	11(4.8%)	5(2.2%)
4-Missed taking for any reason	197(85.7%)	16(7%)	13(5.7%)	4(1.7%)

**Other reasons following the response to question 4:**	**N**	**%**		
Price of medication	11	33.33	33*	
Running out of medication	5	15.15		
Difficult scheduling medication	4	12.12		
Taking around food	1	3.03		
no having choose to take it	2	6.07		
Too many medication to take	3	9.09		
Misplaced medication	5	15.15		
Belief that medication is not working	2	6.06		
**Sum**	33	100		

* Sum of responses of patients that shows non-adherence with other reason.

Logistics correlation test showed that Adherence is more related to the number of transplant (B=1.041, p= 0/048) (Table 3). Also there is significant relationship between medication adherence and quality of life (p≤0.0001, X^2^=12.204) and with health & life performance life (p≤0.0001, X^2^=12.189), and with socioeconomic aspect of quality of life (p=0.003, X^2^=11.322), and with psychological & spiritual dimension life (p=0.017, X^2^=5.669).

**Table 3:** Logistic correlation test

variable	B	df	p- value	Exp(B)
**Age**	0/017	1	0/16	0/98
**Since time of transplant**	0/04	1	0/28	1/041
**number of kidney transplant**	1/04	1	0/048*	2/83

* It shows that adherenceandnumberof kidneytransplant have more relationship.

Todetermine which of dimensions hasmore closely correlation with adherence was used of correlation coefficient ETA.

Results of correlationbetween immunosuppressive medication adherence and Quality of life in this patients shows that there were significant correlation in 3 dimensions of 4: health performance (p≤0.0001 & rETA=0.23), social-economic (p=0.001 & rETA=0.15), psychological- spiritual (p= 0.011 & rETA=0.15)

Medication adherence is most of all related to health/performance dimension life (Table 4).

**Table 4:** Relationship between adherence and dimension’s quality of life

Adherence Quality Of life	rETA	p-value
health - performance	0/23	0/0001*
economic - social	0/15	0/001
psychological - spiritual	0/15	0/011
family	0/18	0/42

* It shows that adherence and health – performance havemore relationship.

## 4. Discussion

Study finding shows there is significant relationship between immunosuppressive medication adherences (ITAS score) and the age of transplant patients with 41-50 age groups according to Table 1, they had the highest adherence. This finding is similar to study of Greenstein and Seagull (1998). They found that elders have more adherence to immunosuppressive therapy relative to younger ones. Furthermore Weng et al. (2013) in therir research found this results, median age 55 (in years) had adherence.

Also, adherence is more related to the time since transplant, i.e. most percentage of adherence is for patients with less than 1 year after transplant and the highest non adherence was among patients whose transplant operation dated back to 16 -21 years ago. This was similar to Greenstein & Seagull study. They found that the more time passes after renal transplant, medication adherence is less probable. It seems that with increasing age of transplant, stress and fatigue occurs in the individual because of continuous follow-ups and as a result the patient would not cooperate to regular medication intake. However, Butler et al. (2004) study did not show any significant relationship between these two variables. Also Burk halter et al. (2014) study showed that Younger age and more years since transplantation were associated with higher non adherence.

Also, there is a significant relation between the number of renal transplant and medication adherence. Present study shows that the people who received their second renal transplant were more non adherence. Percentages in Table 1 points to more adherences to immunosuppressive therapy in first transplant relative to second transplant. Presumably, in patients who are under transplant surgery for the first time are more persistent than patients who have lost kidney in first time of transplant for any reason (including non adherence) Also, medication adherence is related to quality of life in renal transplant patients. In this study according to the result, it seemed adherence has affected on the quality of life because most of the patients who were adherence, had desirable quality of life so immunosuppressant medication adherence is independent variable and quality of life is dependent (Ortega et al., 2009).

Fredricks et al. (2009) in their study on liver transplant adolescents found significant relation between medication adherence and quality of life and depression score and found that non adherence reduces quality of life. Akman et al. (2007) in a study on patients in the renal transplant operation found a significant difference between adherence & quality of life score and depression score. They found that non adhering patients, have lower quality of life and more depression scores. Furthermore, Gorevsk’s study (2013) showed significant relationship between medication non adherence and quality of life, hence kidney transplant patients’ physical functional status was strongly associated with non adherence, and for each point increase in functionality the patients’ adherence increased by 4%.

Also, they noted that non adherence during dialysis might be a similar danger factor for post- renal transplant operation period. Komorosky et al. (2007) found significant difference between heart disease patients and their adherence score and after analysis, it was certain that the low number of drugs, high score of social health and low score of depression and anxiety are predictors of better adherence behavior in heart rehabilitation patients (Chisholm, 2005). Thus, by noting the study results, adherence is an important part of treatment & care that has significant effect on various aspects of quality of life. In this study, more relationship between adherence and health- performance quality of life indicate that immunosuppressive medication adherence have a vital role in improving the performance. Adherence and quality of life have a close relationship and some dimensions are overlap together.

The five dimension related to adherence are social- economic, health care system, condition- related, therapy-related and patient related, so improvement of this 5 dimensions can be helpful and increase medication adherence and ultimately quality of life (Sabate, 2003).

It is suggested that adherence to immunosuppressive therapy should be converted as an essential training in care of these patients. In this study, some patients may have be failure to accept to non adherence or may have problems in recalls so it was better confirmed adherence by serum creatinine concentration. Because of the lack of easy access to patient’slab results therefore it did not.

It is suggested that another research under the same topic should be conducted on heart transplant patients and another one with this same title on a larger sample size.

### 4.1 Limitations

One limitation of our study was that it took place in a single center, though patients were drawn from a variety of ethnic groups and socioeconomic backgrounds, we could not sample from different center because in Tehran there is only one the council of advocacy of renal disorder patients and it was very difficultto accesssamples and more samples. Second limitation was difficulties in access to blood test (blood concentrations of immunosuppressive medication and blood creatinineincrease the accuracy of the measurement non-adherence from participants). In this study was used questionnaire only.

### 4.2 Implications for Nursing and Health Policy

One of the most important health policies is economic implications. Economic implications of non-adherence are increased treatment and nursing care costs alsohealth outcomes that will occur, are diminished quality of life and finally mortality so primary prevention such as education can be effective in decrease non-adherence.

## 5. Conclusion

There was a significant relationship between immunosuppressive medication adherence and quality of life and some patient factors in renal transplant patients. Thus the nurse must take medication adherence as a health enhancement factor while treating and educating these patients.
